# Leadership and Peer Counseling Program: Evaluation of Training and Its Impact on Filipino Senior Peer Counselors

**DOI:** 10.3390/ijerph16214108

**Published:** 2019-10-24

**Authors:** Rogie Royce Carandang, Akira Shibanuma, Junko Kiriya, Karen Rose Vardeleon, Maria Aileen Marges, Edward Asis, Hiroshi Murayama, Masamine Jimba

**Affiliations:** 1 Department of Community and Global Health, Graduate School of Medicine, The University of Tokyo, Tokyo 113-0033, Japan; rrcarandang@gmail.com (R.R.C.); shibanuma@gmail.com (A.S.); jkiriya@m.u-tokyo.ac.jp (J.K.); 2 Institute of Gerontology, The University of Tokyo, Tokyo 113-8656, Japan; murayama@iog.u-tokyo.ac.jp; 3 Childfam-Possibilities Psychosocial Services Co., Quezon City 1104, Philippines; kvardeleon@gmail.com (K.R.V.); mssugusmarges@yahoo.com (M.A.M.); 4 Department of Global Studies, Faculty of Liberal Arts, Sophia University, Tokyo 102-8554, Japan; edward.asis2015@gmail.com

**Keywords:** leadership, senior peer counseling, aging, mental health, Philippines

## Abstract

Senior volunteers represented a significant, mostly untapped lay resource of informal social care. In this study, we evaluated the effectiveness of the training program on improving senior volunteers’ competency toward peer counseling, and explored its impact on their well-being after three months of the program implementation. We conducted a pre- and post-intervention analysis among 60 senior volunteers aged 60–82 years. They participated in 40 h of training and performed weekly peer counseling home visits for three months. We evaluated the program using survey questionnaires, trainer observation and debriefing, and focus group discussions. After the training, peer counselors showed a significant improvement (*p* < 0.001) in knowledge (17.1 versus 22.3) and skills (17.0 versus 17.9). All of them met the minimum required passing level of 70% for the knowledge test, and their overall performance was satisfactory as rated by two independent trainers. After three months, peer counselors showed a significant improvement (*p* < 0.001) in their subjective well-being (*d* = 0.69) and depressive symptoms (*d* = −0.67). Filipino senior volunteers could be trained to serve as peer counselors in their communities. This program improved their competency and well-being. Future research is warranted to determine whether the provision of counseling by them will affect the health outcomes of the target population.

## 1. Introduction

Mental disorders are becoming a major public health threat in the world. Mainly, dementia and depression affect over 20% of adults aged 60 and above, and account for 6.6% of all disability in this age group [1]. However, senior citizens’ mental disorders are underreported [2,3], because their emotional problems were often masked by their physical symptoms [4]. The rate of suicide is also highest among them compared to other age groups [5]. Thus, their mental health needs are serious and must be given attention.

Senior citizens often have unmet needs for mental health. Barriers to treatment include their limited knowledge [6], stigma [7], and lack of access to health professionals [8]. Alternative services are needed to overcome these barriers and address their unmet needs.

The use of an interdisciplinary geriatric team is an effective strategy to deal with the mental health needs of senior citizens [9,10]. The team is usually composed of specialists in mental health, primary care, and rehabilitation working together to integrate care [9,10]. However, in low-resource settings, there is a limited range of public geriatric services and lack of professionals dedicated to mental health [11]. In the Philippines, for instance, there are 0.42 psychiatrists, 0.40 psychiatric nurses, 0.17 medical doctors (not specialized in psychiatry), 0.14 psychologists, 0.08 social workers, and 0.08 occupational therapists per 100,000 members of the general population [12,13]. Faced with these challenges, the government needs innovative strategies to address the emergent needs for mental health among Filipino senior citizens.

Based on existing literature, the ‘stepped care model’ can be used to increase the availability and coverage of mental health services [14]. In this model, the most effective yet less resource-intensive treatment is delivered to patients first, and only stepping up to intensive or specialist services as clinically required [15]. Another strategy is ‘task shifting’, which is the process of delegating tasks to less specialized health workers [16]. This strategy was found useful in low-resource settings [17]. Within this context, the Office for the Senior Citizens Affairs (OSCA) can serve as a focal point for the training of senior volunteers at the paraprofessional level to work as mental health advocates. The OSCA is the institution that is responsible for the planning, implementation, and monitoring of yearly work programs for senior citizens in its target area [18]. It has facilitated various activities such as medical and dental missions, fellowship events, and giving away donations, among others. 

Senior volunteers represented a significant, mostly untapped lay resource of informal social care [19]. They could be adequately trained to serve other disadvantaged senior citizens in the communities [20,21]. They were proven to be effective in group counseling [22], for the blind senior citizens [23], for elderly victims of crime and violence [24], and health education [25]. After the program, peer counselors reported several benefits from their roles, such as an increased sense of well-being and self-worth [26,27,28]. However, most of them also mentioned that their major weakness was the lack of counseling experience [4]. Hence, the integration of ‘leadership’ and didactic and experiential approach to training might empower them to assume their role as peer counselors [29].

Based on the literature, the most effective process for the training of peer counselors include both didactic and experiential techniques [30]. The didactic approach emphasized the shaping of counselor behavior, whereas the experiential approach focused on the counselor’s growth and development [31]. Carkhuff and Truax [32] integrated both methods in the training of both graduate students and lay hospital personnel. They reported that the trainees engaged with their clients almost the same as the more experienced therapists in providing effective psychotherapy. 

In this study, service providers and university investigators worked collaboratively with the local government to develop, implement, and evaluate a leadership and peer counseling program. The program mobilized and trained senior volunteers to become peer counselors. This study aimed to evaluate the effectiveness of the training program on improving senior volunteers’ competency toward peer counseling and to explore its impact on their well-being after three months of the program implementation.

## 2. Methods 

### 2.1. Project Overview and Study Setting

The Project ENGAGE (Embracing and Nurturing Global Ageing) is community-based action research conducted in the City of Muntinlupa from 2017 to 2018. The project had three phases. Phase 1 measured the depressive symptoms [33] and the subjective well-being of community-dwelling senior citizens. Phase 2 trained senior volunteers for leadership and peer counseling, and evaluated the effect of the training and impact of the program. Phase 3 measured the effectiveness of peer counseling and social engagement in improving the well-being of senior citizens at risk of depression. In this paper, we report on the results of Phase 2 of the project following the two aims of our study. First, we compared several outcomes of the senior volunteers before and after the training. Second, we used a mixed-method approach (explanatory design) to explore the impact of the program.

The City of Muntinlupa had nine barangays (communities), and it is the southernmost city in the National Capital Region. It was classified as a highly urbanized city with a poverty incidence of 1.9% in 2012 [34]. The city also has one of the highest records of senior citizens, which account for 5.6% of its population.

### 2.2. Study Participants and Recruitment

We approached the barangay captains to hand over formal invitations for the training to become senior peer counselors. Then, we were referred to the barangay health workers (BHWs) for recruitment, and we organized a meeting with them to discuss the inclusion criteria for potential participants. Individuals were eligible for the study if they were 60 years old and above, registered members of the OSCA, and not engaged in professional mental health care. We excluded senior citizens with moderate or severe cognitive impairment and currently suffering from deafness, aphasia, or other communication disorders. Then, we advised the BHWs to recruit 8–10 senior citizens from each barangay. Senior citizens (n = 70) who were interested visited the barangay hall on the day of the selection interview. We explained the purpose of the study and gathered their training expectations. We selected the senior citizens who showed positive attitudes toward aging, illness, death, adversity, and peer support. After the interview, they were asked to join the training program. Ultimately, 65 senior volunteers met the inclusion criteria and consented to participate in this study. However, only 60 of them completed the 40-h training, while five others dropped out due to conflicts with their work schedule.

### 2.3. Intervention 1: Hands-On Training

Phase 2 of Project ENGAGE aimed to train senior volunteers for leadership and peer counseling. We wanted to build a network of peer counselors who will provide free counseling service to senior citizens at risk of depression in the study area. We held the training from March to April 2018 with the help of a group of mental health experts. They designed the 40-h training program based on their experience and available literature on senior peer counseling [35,36,37]. Table 1 shows the contents of the training provided to senior volunteers. We held a three-day intensive workshop at the OSCA Center and a two-day supervised practicum in the community. We aimed for senior volunteers to achieve the minimum competency to become peer counselors. Trained psychologists, social workers, and BHWs facilitated all activities. We also incorporated a variety of learning modalities, including classroom discussions, group reporting, return demonstration, interactive games, a project proposal, and fieldwork. We divided the participants into two batches by the location’s space and manageability. Each batch consisted of 30 to 35 senior volunteers.

During the supervised practicum, we assigned two clients to each senior volunteer. We recruited the clients from Phase 1 of Project ENGAGE, which previously identified senior citizens who were at risk of depression [33]. We matched senior volunteers and their clients according to shared characteristics such as location, dialect, hobbies, and interests. After completion of the 40-h training, we distributed certificates to 60 senior volunteers, and they were given the title of peer counselors. 

### 2.4. Intervention 2: Peer Counseling Sessions

Following the completion of the training, peer counselors communicated with their assigned clients through weekly home visits for three months. We aimed for peer counselors to meet them for 12 one-hour weekly meetings to identify a client-defined problem, encourage behavior change, build positive relationships, and facilitate connections to community and health services. Peer counselors were required to submit home visitation weekly reports to the trainers to describe their experiences with their clients. Trainers made referrals in cases where the clients needed special social or medical services. We also conducted monthly meetings between peer counselors and trainers to provide emotional and professional support to peer counselors.

### 2.5. Outcome and Assessment

#### 2.5.1. Peer Counselor Knowledge

We assessed peer counselor’s knowledge by self-reported knowledge test. We developed 30-item knowledge questions about leadership and counseling based on the literature [38]. The response options for all the items were ‘yes’ or ‘no’, and possible scores range from 0 to 30. A score of 21 or more indicates that the peer counselor met the minimum passing level of 70%. We performed face validity testing by asking a group of mental health experts before the administration of the questionnaires. 

#### 2.5.2. Peer Counselor Skills

We assessed peer counselor’s counseling skills using an observational scale adapted from a peer educator toolkit [38]. We developed a 22-item checklist that covered eight key skill areas, which include greetings and introduction, asking open-ended questions, showing empathy, helping the client set goals, and so on. A score of 16 or more indicates that the peer counselor met the minimum passing level of 70%. We performed face validity testing by asking a group of mental health experts before the administration of the observational scale. Two trainers were involved during the return demonstration and supervised practicum. We computed the inter-rater reliability between these two trainers after data collection. We also debriefed after each training session. 

#### 2.5.3. Peer counselor Satisfaction

We evaluated peer counselors’ training satisfaction using rating scale questions [4,39], which displayed a scale of either from ‘1 to 5’ or ‘0 to 10’. Peer counselors were asked to select the numerical point on the scale that represents their response best. The higher the numerical point, the more they strongly agree with the statement, and vice versa. We performed face validity testing by asking a group of mental health experts before the administration of the rating scale. The Cronbach’s α for this study was 0.86.

#### 2.5.4. Peer Counselor Mental Health Status

We measured the depressive symptoms of peer counselors by the 15-item Geriatric Depression Scale (GDS-15). This scale was specially developed for use in geriatric patients and contained fewer somatic items [40,41]. The response options for all the items were ‘yes’ or ‘no’, and possible scores range from 0 to 15. A score of 5 or more indicated a tendency toward depression. The validity and reliability of GDS-15 have been supported through both clinical practice and community-based research [42,43]. The Cronbach’s α for this study was 0.84.

We assessed the subjective well-being of peer counselors using the 5-item World Health Organization (WHO) Well-Being Index (WHO-5). The WHO-5 is a short scale for the measurement of positive well-being [44]. It consisted of five positively phrased items that measure the participant’s well-being over the last two weeks. This scale provided six-point Likert response options ranging from 0 (at no time) to 5 (all of the time). The total score was the sum of the five items ranging from 0 to 25, where 0 represented the worst possible quality of life, and 25 represented the best possible quality of life. A score below 13 indicated poor well-being, and is an indication for further evaluation. The Cronbach’s α for this study was 0.88. 

#### 2.5.5. Peer Counselor Training and Client Experience

We conducted eight Focus Group Discussions (FGDs) during disengagement and each FGD comprised of 6–8 members. We used a semi-structured questionnaire to evaluate peer counselors’ training and client experience. All peer counselors participated in the FGDs. Areas of inquiry included thoughts and feelings about the training experience, the group environment, the counseling experience, and perceptions of themselves as peer counselors. FGDs lasted for 60–90 min; they were digitally recorded, transcribed verbatim, and analyzed thematically.

### 2.6. Data Collection

We administered a pre-training survey to measure the baseline knowledge of peer counselors. We used the observation ratings from return demonstration as their baseline skills, because senior volunteers had no prior experience in counseling. We also measured their baseline depressive symptoms and subjective well-being. After the training, we conducted post-training surveys for knowledge, skills, and peer counselor satisfaction. Three months later, we conducted a post-intervention survey of their mental health status. All peer counselors completed the questionnaires in less than 30 min. We used ID numbers to match the peer counselor’s pre- and post-intervention responses. For the qualitative data, we conducted eight FGDs among peer counselors (each FGD comprised of 6–8 members) to elicit perceptions of the program and know their experiences with their clients. 

### 2.7. Data Analysis

We calculated the total scores for knowledge, skills, peer counselor satisfaction, GDS-15, and WHO-5. We conducted a paired-sample *t*-test, and calculated the effect size to compare peer counselors’ competency scores and mental health status pre- and post-intervention. We calculated Cohen’s kappa coefficient to examine the inter-rater reliability of the observational scale between two trainers. We set the level of significance at 0.05 (two-tailed) and performed statistical analyses using Stata 13.1 (StataCorp, College Station, TX, USA). For qualitative data (FGDs), we examined the impact of the program on peer counselors using thematic analysis [45,46]. Two authors (R.R.C. and E.A.) read and coded the transcripts and categorized all codes independently. We identified themes by relating categories and subcategories. We validated the coding and themes through continuous dialogue among co-researchers and peer debriefing to interpret results with psychologists [47]. We also conducted a ‘member check’ by providing feedback to the peer counselors. We gave them a summary of the findings before finalizing the themes and categories. They validated our results and gave valuable comments to refine our findings further. After reaching an agreement, we translated the identified themes and quotations to support themes into English.

### 2.8. Ethical Considerations

The Project ENGAGE was approved by the University of Tokyo Research Ethics Committee (SN 11641) and the University of the Philippines-Manila Research Ethics Board (UPMREB 2017-312-01). We ensured the confidentiality of the peer counselors’ responses, and their privacy was strictly protected as no personally identifiable information was used in this study. We secured written informed consents before the training, and all participation was voluntary. Also, peer counselors were free to withdraw from the project at any time, for any reason, without penalty. 

## 3. Results

### 3.1. General Characteristics of Participants

Table 2 shows the socio-demographic characteristics of peer counselors. Of the 60 peer counselors, 95.0% (n = 57) were women, and their mean age was 67.3 years [standard deviation (SD) 5.6], and that of men was 66.3 (SD 4.7). Half of them reported as being widowed and no source of income. In addition, nearly half (46.7%) did not have a pension. All of the peer counselors had a secondary/tertiary education. Concerning their general health status, half of them (51.7%) reported having a ‘fair’ health condition, and most of them (86.7%) already had at least one chronic disease. For their living arrangement, 54 (90.0%) of them lived with others. The majority of them were never-smokers (49, 81.7%) and non-drinkers (52, 86.7%).

### 3.2. Outcome Evaluation

#### Peer Counselor Competency

Table 3 shows the comparison of peer counselors’ pre- and post-intervention competency scores. All peer counselors met the minimum required passing level of 70% for the knowledge test, with a mean knowledge score of 74.3% (based on 100 as a perfect score). Endpoint analyses showed that the training significantly increased the knowledge score of peer counselors (mean score at baseline 17.1, SD 2.5, versus mean score after the training 22.3, SD 2.4) (*t* = 12.89, df = 59, *p* < 0.001). Concerning skills, independent observational ratings by two trainers revealed that peer counselor performance was at a satisfactory level (return demonstration score 77.2%; supervised practicum score 81.4%). Endpoint analyses showed that the practicum significantly improved the skills score of peer counselors (mean score at baseline 17.0, SD 3.0, versus mean score after the practicum 17.9, SD 2.8) (*t* = 6.96, df = 59, *p* < 0.001). As for effect size, the training had a large effect on knowledge (*d* = 1.66) and skills (*d* = 0.90). Inter-rater reliability between the two trainers showed substantial agreement (return demonstration: kappa 0.72, *p* < 0.001; supervised practicum: kappa 0.74, *p* < 0.001), indicating good inter-rater reliability.

### 3.3. Process Evaluation

#### Peer Counselor Satisfaction

Table 4 shows the training evaluation of peer counselors. The results indicated a high level of satisfaction among them with the overall program (average 9.7 on a 10-point scale) and with specific aspects of the program, which include topics chosen, time allocation, training materials, trainer quality, and training facility (range of item means = 4.2–4.5 on a 1–5 scale). Moreover, peer counselors found the training relevant, useful, and easy to understand, which might have led to an increased amount of knowledge and change in their attitude (range of item means = 9.4–9.6 on a 0–10 scale).

### 3.4. Impact Evaluation

#### 3.4.1. Peer Counselor Mental Health Status

Table 5 shows the comparison of peer counselors’ pre- and post-intervention mental health status. After the three-month intervention, the mean total GDS-15 scores decreased significantly (at baseline 4.4, SD 2.4, versus after the program, 2.9, SD 2.2) (*t* = −5.21, df = 59, *p* < 0.001), while the mean total WHO-5 scores increased significantly (at baseline 15.3, SD 4.2, versus after the program 19.1, SD 4.2) (*t* = 5.32, df = 59, *p* < 0.001). As for effect size, the three-month program showed a medium effect on subjective well-being (*d* = 0.69) and depressive symptoms (*d* = −0.67). Of the 60 peer counselors, two of them experienced a worsening of mental health status after participating in the three-month program.

#### 3.4.2. Peer Counselor Training and Client Experience

Table 6 shows the impact of the program on peer counselors that emerged from the thematic analysis of qualitative data. The FGDs with peer counselors showed three significant themes: (1) personal growth, (2) opportunities as a peer counselor, and (3) challenges as a peer counselor. We also showed example quotes in Table 6.

As for personal growth, peer counselors mentioned improvement in their knowledge, interpersonal skills, social relations, and awareness of self and others. These were all attributed to their training experience. When asked what they liked most about the training, they talked about learning new concepts, interacting with fellow senior citizens, and the supportive nature of both the participants and trainers. 

Concerning opportunities as a peer counselor, four sub-themes emerged: advising, recognition, companionship, and active lifestyle. They mentioned that advising played a significant role in their whole journey as peer counselors. Recognition, on the other hand, was a fruit of their passion for helping others in their community. Many of them said that they had found companionship with their clients and fellow peer counselors. They also said that the program kept them active and gave them a chance to go outdoors.

However, some negative feedback was received, which was mainly about the challenges faced by peer counselors. These include dealing with difficult and indigent clients, as well as finding a balance between health and counseling duties. 

## 4. Discussion

In this study, we demonstrated that Filipino senior volunteers could be trained to serve as peer counselors in their community. The 40-h training showed significant improvement in peer counselors’ competency. Their competency scores indicated that they were ready to assume their role as peer counselors. Their training evaluation also revealed that they were satisfied with the training they received and felt empowered to do their job. After the three-month program, peer counselors showed improvement of mental health status. 

The success of the training program can be attributed to the didactic and experiential approach [31,48,49], which consists of four essential elements in this study. First, the three-day intensive workshop gave the peer counselors the common base of knowledge and skills that they applied during the two-day practical training in the community. Second, the leadership training empowered peer counselors to demonstrate their leadership potential and to build strong relationships with their peers. The team building activities encouraged them to work as a group and take action toward healthy aging. Third, the return demonstration allowed them to practice their skills with supervision and feedback until they achieved minimum competency. Then, peer counselors exhibited satisfactory results in their communication and counseling skills. Finally, the supervised practicum in the field allowed peer counselors to practice their acquired skills in real counseling situations. The fieldwork allowed them to identify vital areas that need to be further improved. However, we noted areas needing improvement. These included the handling of questions, reflecting the client’s feelings, helping the client set goals, and summarizing each counseling session.

The leadership and peer counseling program had an impact on the well-being of peer counselors. After the three-month program, peer counselors showed statistically significant improvement in depressive symptoms and subjective well-being. Results of the qualitative analysis suggested some contributing factors that can explain such significant change. The most strongly suggested factor was personal growth, which can be attributed to the training experience. In this study, peer counselors expressed an improvement in knowledge, interpersonal skills, social relations, and awareness of self and others. These findings were consistent with those reported about participants in other senior peer counseling programs [24,26,27]. Other factors that might contribute to the improvement of their mental health are the opportunities gained by the peer counselors during the three-month program. For instance, volunteer work allowed them to remain actively engaged in their communities. Being able to give advice and help clients who were in need contributed to their sense of purpose. Moreover, community recognition of their role as a peer counselor enriched their self-esteem, while companionship with their peers and clients extended their social network. Butler [50] also highlighted the benefit of companionship with clients in their senior companion program.

Although the present findings suggested that the client experience may improve the well-being of peer counselors, this must be interpreted with caution. Peer counselors in this study also reported some challenges with their clients. These include dealing with difficult and indigent clients, as well as finding a balance between health and counseling duties. These challenges may harm the well-being of peer counselors. Denton et al. [51] previously emphasized that workload and difficult clients were associated with poorer health among home care workers.

We acknowledged several limitations of this study. First, the scope of the training program did not allow us to assign participants to treatment and control groups. Thus, we cannot attribute the outcomes to specific intervention components or rule out other factors. Second, peer counselors were predominantly women. The gender-based differences may have affected their working alliance with male clients [52]. Third, the sample size was small (n = 60), and the peer counselors were recruited from one particular city in the Philippines. Hence, expansion of the project to the rural areas will provide further information. Finally, some of the measures such as knowledge and skills assessment were adapted from previous studies [4,38,39], and have not been validated yet in the Filipino context. To overcome this, we did forward and back translations carefully, and performed face validity testing by asking a group of mental health experts before the administration of the questionnaires. Regardless of the limitations, the findings from this study highlighted the potential for peer counselors to provide services to at-risk and underserved senior citizens. Given the limited human resource for formal health care in the Philippines, the involvement and integration of volunteers could also be expanded in the community.

## 5. Conclusions

In this study, we demonstrated that senior volunteers could be trained to serve as peer counselors in their communities. We equipped them with the proper knowledge, skills, and attitude to assume leadership roles in conducting peer counseling. Moreover, this program has the potential to improve their well-being. Essential factors that might have made this program effective were the didactic and experiential approach in training, coupled with the supportive nature of the trainers. Future research is warranted to determine whether the provision of peer counseling by them will affect the health outcomes of the target population. Other low-resource communities might also benefit from the Philippines’ leadership and peer counseling program, and train their senior volunteers in similar ways. As for the recommendation, peer counselors should find balance and seek constant supervision from their trainers. More male peer counselors should also be involved in the program to address gender-based differences. In the future, similar programs should be implemented, and their effectiveness examined both by process and outcome measures.

## Figures and Tables

**Table 1:** Overview of training for leadership and peer counseling provided to peer counselors.

Training Settings	Total Hours	Topics Covered
Three-day intensive workshop	24	Course overview and introduction to the training Roles and responsibilities of senior peer counselors Aging and mental health Leadership skills Building strong relationships Ability to take action toward healthy aging Communication and counseling skills Psychosocial support Positive living Record keeping and reporting Making of a community-based project
Two-day practical training in the community	16	Supervised practicum (fieldwork) focusing on the following skills: Greetings and introduction Using helpful nonverbal communication Asking open-ended questions Listening actively and showing interest in the client Reflecting what the client is saying Showing empathy, not sympathy Avoiding judging words Helping the client set goals and summarizing each counseling session

**Table 2:** Socio-demographic characteristics of peer counselors.

Characteristics	Total (n = 60)	
	n	%
Age, mean (SD)	67.3 (5.5)	
Sex		
Male	3	5.0
Female	57	95.0
Marital status		
Married	26	43.3
Never married	3	5.0
Separated	1	1.7
Widowed	30	50.0
Education		
Secondary/Tertiary	60	100.0
Monthly income		
No income	33	55.0
Poor income	16	26.7
Average/Good income	11	18.3
Pension		
Have	32	53.3
Do not have	28	46.7
Self-rated health		
Good/Very good	18	30.0
Fair	31	51.7
Bad/Very bad	11	18.3
Chronic diseases		
Have	52	86.7
Do not have	8	13.3
Living arrangement		
Alone	6	10.0
Living with others	54	90.0
Smoking		
Never smoker	49	81.7
Ex-/Current smoker	11	18.3
Drinking alcohol		
Non-drinker	52	86.7
Occasional/ Daily drinker	8	13.3

Note: SD—Standard deviation.

**Table 3:** Comparison of peer counselors’ pre- and post-intervention competency scores.

Measures	Pre-Intervention	Post-Intervention	*t*-Value	*p*-Value	Effect Size ^a^
Peer counselor competency	Mean (SD)	Mean (SD)			
Leadership and counseling knowledge	17.1 (2.5)	22.3 (2.4)	12.89	<0.001	1.66
Observational ratings (skills)	17.0 (3.0)	17.9 (2.8)	6.96	<0.001	0.90

Note: SD—Standard deviation; ^a^ This effect size (Cohen’s *d*) is a standardized measure of the difference between after and before the intervention in standard-deviation units.

**Table 4:** Training evaluation of peer counselors.

Measure	Range (Possible Range)	Mean	SD
Peer counselor satisfaction			
Topics chosen	3–5 (1–5)	4.4	0.8
Time allocation	3–5 (1–5)	4.3	0.8
Training materials	3–5 (1–5)	4.4	0.8
Trainer quality	3–5 (1–5)	4.2	0.9
Training facility	3–5 (1–5)	4.5	0.7
Clarity and understandability	6–10 (0–10)	9.4	1.1
Amount of new knowledge gained	7–10 (0–10)	9.6	0.9
Relevance and usefulness	7–10 (0–10)	9.5	0.9
Amount of attitude change	7–10 (0–10)	9.6	0.9
Overall satisfaction	7–10 (0–10)	9.7	0.7

Note: SD—Standard deviation.

**Table 5:** Comparison of peer counselors’ pre- and post-intervention mental health status.

Measures	Pre-Intervention	Post-Intervention	*t*-Value	*p*-Value	Effect Size ^a^
Peer counselor mental health	Mean (SD)	Mean (SD)			
Depressive symptoms (GDS-15)	4.4 (2.4)	2.9 (2.2)	−5.21	<0.001	−0.67
Subjective well-being (WHO-5)	15.3 (4.2)	19.1 (4.2)	5.32	<0.001	0.69

Note: SD—Standard deviation; ^a^ This effect size (Cohen’s *d*) is a standardized measure of the difference between after and before the intervention in standard deviation units.

**Table 6:** Impact of leadership and peer counseling program on peer counselors’ well-being.

Key Themes	Sub-Themes	Example Quotes
Personal growth	Improved knowledge Improved awareness of self and others	▪ *‘Everything was interesting, and I felt delighted after the training. I noticed a big change within me. I have this enthusiasm to learn something new, and I became more aware of myself and others. The training empowered me to assume my role as a peer counselor.’* (female peer counselor)
	Improved interpersonal skills Improved social relations	▪ *‘I was shy and not into sharing my personal life. However, the training helped me build confidence and improved my communication skills. I have found new friends in our training. All the participants and trainers were very supportive, and I feel comfortable being around with them.’* (male peer counselor)
Opportunities as a peer counselor	Advising Recognition	▪ *‘I recognized myself as an effective peer counselor when my clients took my advice and changed their behavior. I had a client who is alcoholic and unemployed. I wanted him to be more productive, so I gave him the capital to start a small business (selling rags). Now, the business is going well. I felt glad to make life better for seniors in need.’* (female peer counselor) ▪ *‘The entire program was new in our community, and people were surprised to know that counseling exists. Through my weekly home visits, people in my neighborhood started to recognize me as a peer counselor. It felt like I got a new role in our community. They were all interested in joining our program.’* (female peer counselor)
	Companionship	▪ *‘We have formed a support network among ourselves. We turn to each other for fun and help. There was a time I got sick, and they visited my place to show their concern. I felt like I was part of a large extended family.’* (female peer counselor) ▪ *‘My clients and I shared our own difficult life experiences, and this lead to building mutual trust. I made my clients feel that we were on the same level. Together, we think of ways of solving our problems.’* (female peer counselor)
	Active lifestyle	▪ *‘Before joining the program, I spent most of my days taking care of my grandchildren. Working as a peer counselor allows me to go to OSCA and visit my clients once a week. Going outdoors keeps me active, which is good for my health.’* (female peer counselor)
Challenges as a peer counselor	Difficult clients	▪ *‘I only had my first visit with one of my clients. At first, he welcomed me, but during my second visit, he asked me to leave. I was not sure what went wrong. His wife told me that my client does not want to talk to anyone. I find it difficult to deal with that male client.’* (female peer counselor)
	Indigent clients	▪ * ‘My client is very sick and poor. I feel sorry to watch him in pain. He was hoping to receive financial aid from our program. To help, I always bring bread during my visits to him.’* (male peer counselor)
	Finding balance	▪ *‘It was summer when we started the program. The hot weather triggered my hypertension. I cannot join my fellow peer counselors during weekly home visits. I have to put off my counseling duties and wait until I recover from my illness.’* (female peer counselor)

Note: OSCA—Office for the Senior Citizens Affairs.
